# Characterization of the biological processes shaping the genetic structure of the Italian population

**DOI:** 10.1186/s12863-015-0293-x

**Published:** 2015-11-09

**Authors:** Silvia Parolo, Antonella Lisa, Davide Gentilini, Anna Maria Di Blasio, Simona Barlera, Enrico B. Nicolis, Giorgio B. Boncoraglio, Eugenio A. Parati, Silvia Bione

**Affiliations:** Computational Biology Unit, Institute of Molecular Genetics-National Research Council, Pavia, Italy; Molecular Biology Laboratory, Istituto Auxologico Italiano, Milan, Italy; Department of Cardiovascular Research, IRCCS Mario Negri Institute for Pharmacological Research, Milan, Italy; Department of Cerebrovascular Diseases, IRCCS Istituto Neurologico Carlo Besta, Milan, Italy

**Keywords:** Latitude, Immunity, Pathogen, LincRNA

## Abstract

**Background:**

The genetic structure of human populations is the outcome of the combined action of different processes such as demographic dynamics and natural selection. Several efforts toward the characterization of population genetic architectures and the identification of adaptation signatures were recently made. In this study, we provide a genome-wide depiction of the Italian population structure and the analysis of the major determinants of the current existing genetic variation.

**Results:**

We defined and characterized 210 genomic loci associated with the first Principal Component calculated on the Italian genotypic data and correlated to the North–south genetic gradient. Using a gene-enrichment approach we identified the immune function as primarily involved in the Italian population differentiation and we described a locus on chromosome 13 showing combined evidence of North–south diversification in allele frequencies and signs of recent positive selection. In this region our bioinformatics analysis pinpointed an uncharacterized long intergenic non-coding (lincRNA), whose expression appeared specific for immune-related tissues suggesting its relevance for the immune function.

**Conclusions:**

Our study, combining population genetic analyses with biological insights provides a description of the Italian genetic structure that in future could contribute to the evaluation of complex diseases risk in the population context.

**Electronic supplementary material:**

The online version of this article (doi:10.1186/s12863-015-0293-x) contains supplementary material, which is available to authorized users.

## Background

Understanding the genetic structure of human populations is crucial to reconstruct their history and to elucidate the genetic predisposition to diseases. In fact, the genetic structure of human populations was shaped by several demographic events and selective forces, which have contributed to the current diversification and to the differences in diseases prevalence and predisposition [1, 2]. Some relevant examples highlighting the relationship between migration, selection and disease were recently reported, like the gradient in type 2 diabetes genetic risk moving out of Africa [3] or the demonstration that common risk alleles for inflammatory diseases are targets of recent positive selection [4]. Therefore, the study of the genetic architecture of common disorders requires a deep knowledge of the dynamics affecting the population under investigation.

In recent years, the genetic structure of several human populations has been characterized both at worldwide and regional level using genome-wide markers. In Europe, the genetic variation pattern showed a southeast-northwest gradient with a strict correspondence between genetic and geographic distances [5–7]. Along the European latitudinal gradient, Italy plays a major role due to its central position and its geographical conformation extended in the Mediterranean area. The genetic structure of the Italian population has been explored since a long time, starting from pioneering studies based on classic genetic markers [8], to recent works involving genome-wide approaches [9]. Altogether these studies demonstrated the presence of a North–South gradient in allele frequencies along the peninsula and the differentiation of Sardinia from the mainland. The observed European latitudinal cline in allele frequencies has been interpreted as the consequence of human migrations since Paleolithic [10].

In addition to demographic processes, several evidence of positive selection differentially shaping the genome of human populations have been described [11, 12]. In the European population, the best known signature of adaptation is represented by the lactase gene (*LCT*) which confers ability to digest lactose in adulthood. The lactase persistence shows a latitudinal cline with particularly high rates among Northern Europeans and it was demonstrated to be a target of natural selection [13]. Moreover, weak polygenic adaptation acting on many loci at the same time and slightly modifying allele frequencies has been also described as a shaper of human diversity [14]. As an example, human height, a polygenic highly heritable trait, has been proposed as a target of widespread selection on standing variation resulting in differences in adult height between northern and southern European populations [15].

Although the genetic structure of different populations has been deeply characterized, the underlining biological processes are still poorly understood thus requiring further investigations, both at worldwide and regional level.

In this paper, we exploited genome-wide genotypic data to recapitulate the genetic structure of the Italian population in its geographic context, refining the picture of the North–South gradient in genetic variation. A total of 210 genomic loci, sufficient to explain the latitudinal cline in genetic variation, were identified and characterized by different bioinformatics approaches.

## Results

### The genetic structure of the Italian population

To investigate the genetic structure of the Italian population we assembled a genome-wide genotype dataset of 1736 Italian individuals, as detailed in the Methods section.

**Fig. 1:**
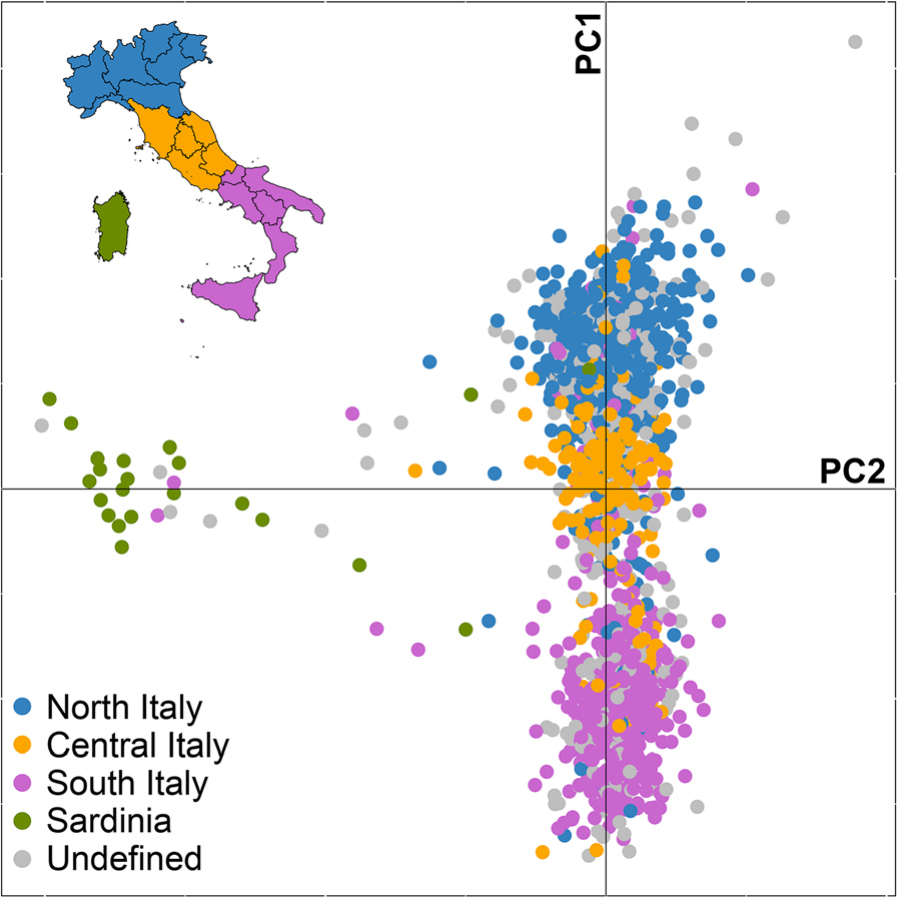
Principal Component Analysis of the Italian population. Plot of the first two principal components calculated on the Italian genotypic dataset. Each individual was labeled according to the color scheme reported in the map in the upper-left corner. The map of Italy was created using the shapefile made available by the Italian National Institute of Statistics (ISTAT; http://www.istat.it/it/strumenti/territorio-e-cartografia)

After a quality-control procedure, the Italian genetic diversity was summarized by Principal Component Analysis (PCA) using the *smartpca* tool of the EIGENSOFT package [16]. To gain insight into the observed differentiation and test the existence of positive correlation with geography we assigned a geographic place of origin to the individuals through the analysis of their surnames (see Methods and Additional file 1). We observed that the clustering of individuals obtained from the plot of the first two Principal Components (PCs) reflected the geographical origin of each individual obtained from the surname analysis (Fig. 1). In particular, the first principal component (0.17 % of total variance explained) showed a North–South gradient that well correlated with latitude (Pearson’s correlation coefficient *r* = 0.876, *p* = 8.805 × 10^−7^). The regional subdivision of the Italian population was also evaluated using the pairwise F_ST_ parameter as a measure of genetic distance. A significant correlation between the matrix of F_ST_ and the matrix of the kilometric distances between regional capitals was found (Mantel test, *z* = 59.7, *p* = 3.499 × 10^−5^). The second principal component (0.09 % of total variance explained) differentiated Sardinian individuals from the others, reflecting their known genetic diversity. The other PCs did not show any correlation with the Italian geography.

We also evaluated the Italian genetic diversity in the surrounding geographic context through the analysis of available genotype data from populations of the European and Mediterranean area (Additional file 2). The PCA and the ancestry estimation method implemented in ADMIXTURE [17] revealed that Italy stood at the crossroad between continental Europe and the Mediterranean region thus confirming the North–South gradient previously described (Additional file 3, 4, 5).

### Genomic loci contributing to latitudinal cline in the Italian population

To evaluate the involvement of specific biological processes in the North–South differentiation of the Italian population, we investigated the genetic variants contributing to PC1.

**Fig. 2:**
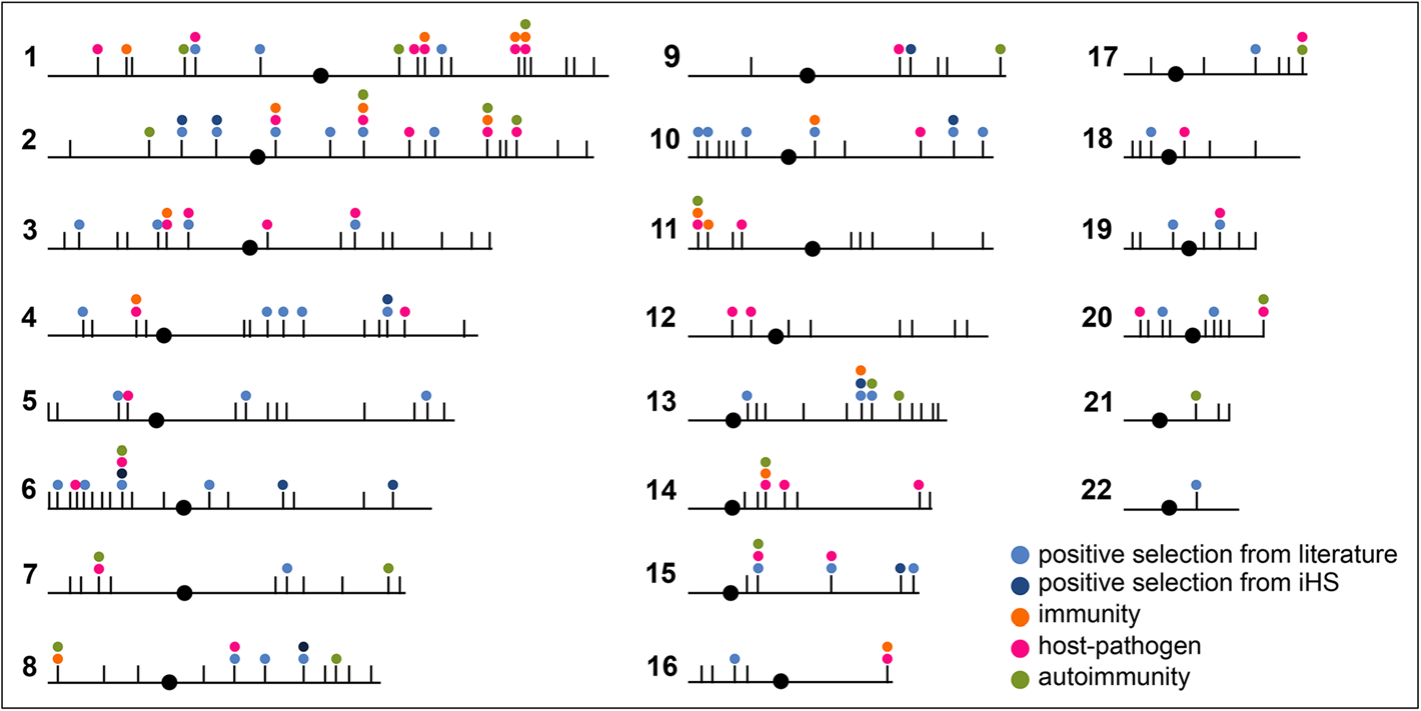
Genomic distribution of the 210 Italian PC1-associated loci. Chromosomes were represented as horizontal straight lines with centromeres represented as black circles. The vertical dashes correspond to the 210 loci. The circles above the loci were colored based on positive selection features and functional annotation of the contained genes according to the legend in the lower-right corner

Through a linear regression analysis, after applying a genome-wide *p*-value threshold of 1 × 10^−7^, we identified a total of 270 SNPs significantly associated with PC1 and sufficient to recapitulate the Italian latitudinal cline (Additional file 6). On the basis of linkage disequilibrium (LD) features of the genomic regions where the single nucleotide polymorphisms (SNPs) were located, we defined a total of 210 loci contributing to the North–South gradient (Fig. 2 and Additional file 7). The identified loci covered a total of 74.5 Mb, they were on average 355 kb wide (range: 10 kb–2.4 Mb) and were distributed along all autosomes. Thirteen loci appeared devoid of any transcribed regions whereas the remaining contained 702 RefSeq genes, with an average of 3.3 gene/locus. According to the HUGO Gene Nomenclature Committee (HGNC) classification [18], 82 % of genes were protein coding (*n* = 578), 14 % were non-coding RNAs (*n* = 99) and the remaining 4 % were pseudogenes (*n* = 25). When we tested the enrichment in gene content of the 210 genomic intervals, a slight overrepresentation was observed (*p* = 0.0595) and it resulted statistically significant considering only the protein-coding genes (*p* = 0.0014). Moreover, the enrichment in genes causing Mendelian diseases resulted significant (*p* = 0.0223). Using the National Human Genome Research Institute (NHGRI) Genome Wide Association Study (GWAS) catalogue [19], we found that 475 genetic variants involved in the predisposition to common disorders were located in the Italian PC1 loci (*p* = 0.0126).

To evaluate the involvement of the 702 genes in specific biological functions, we performed a gene-sets enrichment analysis. The human leukocyte antigen (HLA) region was excluded from the analysis, since it harbors several genes with known immune functions. The overrepresentation of Gene Ontology (GO) terms was evaluated using MSigDB of the Gene Set Enrichment Analysis (GSEA) package (see Methods) [20]. 8 GO terms resulted significantly enriched in the “Biological process” category (Table 1 and Additional file 8). Among them, the GO term “Signal transduction” resulted as the most enriched, indicating the presence of an high number of genes involved in cell function regulation. The second most significant GO term was “Regulation of cellular metabolic process”. Moreover, the GO term “Immune system process” resulted significantly overrepresented and it contained seven genes (*IL6*, *CHUK*, *CXCR4*, *CD79A*, *CNR2*, *FCGR2B* and *MAL*) in common with the “Signal transduction” term and four genes (*IL6*, *CHUK*, *HDAC4* and *CEBPB)* shared with the “Regulation of cellular metabolic process” clade pointing out an overall interconnection among these biological processes. Taking into account the “Cellular Component” ontology, “Membrane” resulted as the most significantly enriched term together with 7 other terms referring to the membrane portion of the cell (Table 2 and Additional file 8). None of the “Molecular function” GO terms resulted enriched below the defined threshold. The analysis of canonical pathways, performed with the Ingenuity Pathway Analysis (IPA) tool, identified “Role of NFAT in Regulation of the Immune Response” as the most enriched pathway. Interestingly, other pathways were related to the immune response processes, underlining the relevance of this biological function in the Italian population PC1-associated gene list (Table 3 and Additional file 8).

**Table 1:** Significantly enriched GO Biological Processes

GO term ID	GO term name	# Genes	FDR *q*-value
GO:0007165	Signal transduction	53	2.66E-07
GO:0031323	Regulation of cellular metabolic process	31	1.00E-05
GO:0019222	Regulation of metabolic process	31	1.00E-05
GO:0006139	Nucleobase-containing compound metabolic process	40	1.36E-05
GO:0002376	Immune system process	18	4.10E-05
GO:0043283	Biopolymer metabolic process	47	4.10E-05
GO:0019219	Regulation of nucleobase-containing compound metabolic process	25	4.20E-05
GO:0010468	Regulation of gene expression	26	5.14E-05

**Table 2:** Significantly enriched GO Cellular Components

GO term ID	GO term name	# Genes	FDR *q*-value
GO:0016020	Membrane	72	1.78E-13
GO:0005886	Plasma membrane	55	2.27E-11
GO:0005737	Cytoplasm	65	2.85E-09
GO:0044425	Membrane part	55	4.23E-09
GO:0044459	Plasma membrane part	44	4.23E-09
GO:0031226	Intrinsic to plasma membrane	38	5.48E-08
GO:0031224	Intrinsic to membrane	45	9.45E-08
GO:0005887	Integral to plasma membrane	37	9.45E-08
GO:0016021	Integral to membrane	44	1.61E-07

**Table 3:** Significantly enriched IPA Canonical Pathways

IPA pathway	# Genes	Ratio	*p*-value
Role of NFAT in Regulation of the Immune Response	14	0.082	2.40E-04
Glutamate Receptor Signaling	7	0.123	8.32E-04
Phagosome formation	10	0.09	8.71E-04
Dendritic Cell Maturation	13	0.073	1.20E-03
Synaptic Long Term Depression	11	0.077	1.70E-03
Protein Kinase A Signaling	21	0.054	2.04E-03
JAK/Stat Signaling	7	0.097	3.31E-03
TREM1 Signaling	7	0.093	4.17E-03
CREB Signaling in Neurons	11	0.064	6.92E-03
Chondroitin Sulfate Degradation (Metazoan)	3	0.2	7.08E-03

### Impact of natural positive selection on the 210 Italian PC1 loci

Signals of positive selection were identified using the integrated haplotype score (iHS) statistics [21], which was calculated for each of the autosomal SNPs. We grouped SNPs into non-overlapping genomic intervals of 200 kb: the proportion of SNPs with an |iHS| greater than or equal to 2 was calculated for each interval and those lying in the 5 % tail of the resulting distribution were considered as significant. This approach resulted in the selection of 509 genomic intervals. The intersection between the PC1-associated loci and the iHS significant windows resulted in the identification of 17 loci harboring both signals, thus highlighting the contribution of selection in the Italian North–South differentiation.

Additional insights into the contribution of positive selection as a mechanism involved in the determination of the Italian North–South genetic gradient were obtained by comparison with literature data and testing the enrichment in gene lists for biological functions known to be target of positive selection. Taking into account recent publications based on genome-wide genotypic data and performed on different populations [11, 22, 23], a total of 47 Italian PC1-associated loci was found to overlap at least one genomic interval for which evidence of positive selection were demonstrated (Fig. 2). Eight of the 17 loci identified using the iHS parameter were previously described as targets of positive selection by different studies (Fig. 2 and Additional file 7). When we evaluated loci enrichment for sets of gene involved in skin pigmentation, immunity, response to infectious disease, sensory perception and metabolism, previously defined by Grossman et al. [23], we found a significant result for immunity and pigmentation (respectively INRICH target-test *p* = 0.017 and *p* = 0.004).

**Fig. 3:**
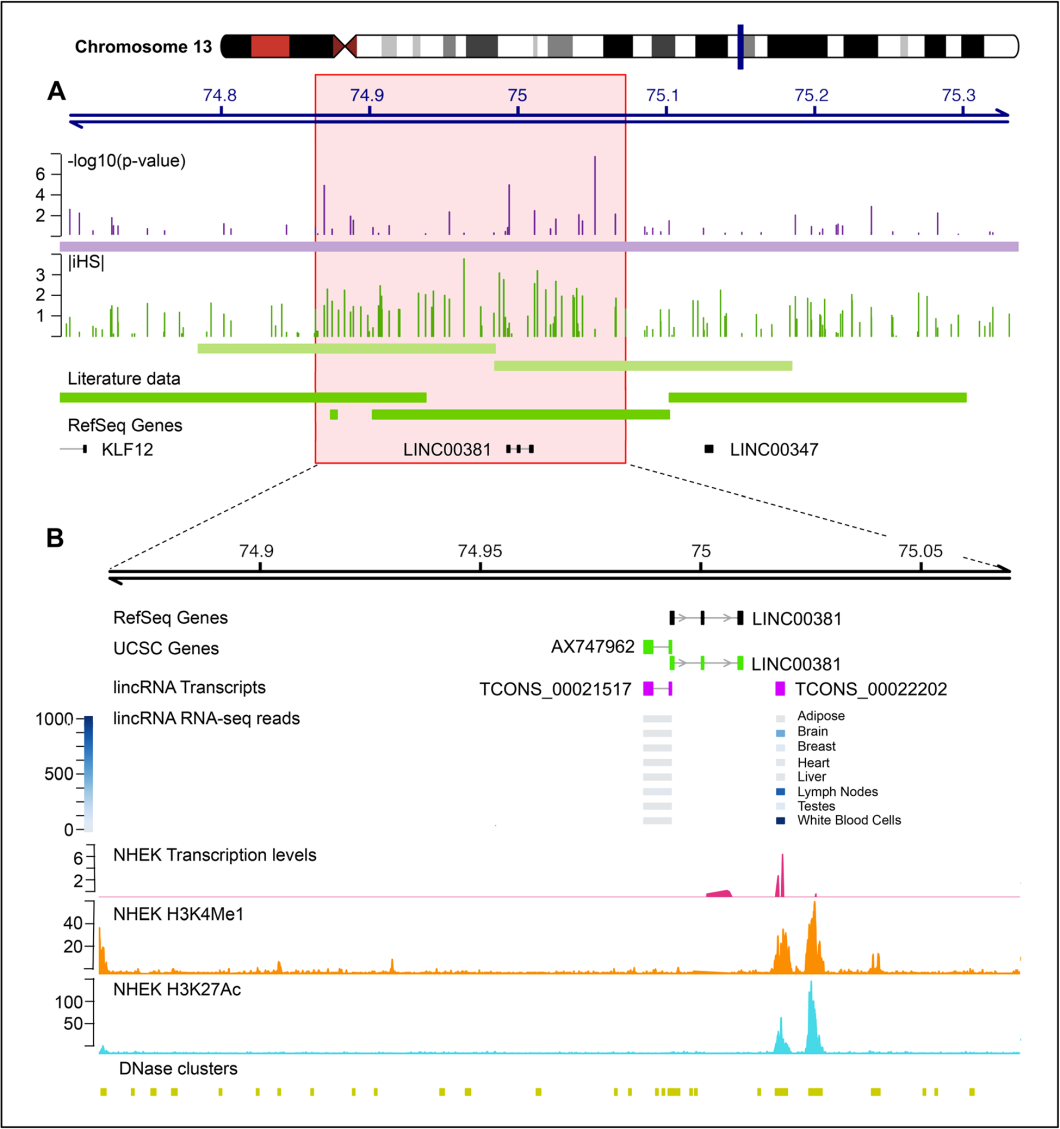
Characterization of the newly identified locus at 13q22.1. **a** Below the line representing base positions in Mb, different features were represented: the PC1 association signals (*vertical dark violet lines*), the |iHS| value for each SNP tested (*vertical dark green*), the 200 kb intervals defined as positively selected according to the iHS analysis (*light green bars*), the genomic intervals with evidence of positive selection from the literature (*darker green bars*) and RefSeq genes (*black lines*); **b** detail of the core region defined showing: RefSeq genes (*black*), UCSC genes (*green*), lincRNA transcripts (*purple*) and lincRNA RNAseq reads (*blue scale*) according to Cabili et al. 2011 [25], transcription levels and epigenetic features in NHEK cell line from the ENCODE project, the DNase hypersensitivity clusters from 125 ENCODE cell types

In particular, the locus on chromosome 13 at nucleotide position 74,690,999–75,337,499 (locus 155 in Additional file 7) resulted to contain two intervals with a significant proportion of SNPs with |iHS| > = 2 and to overlap to selection signals previously identified by analyses performed on the HapMap and Human Genome Diversity Projects (HGDP) populations [11, 22, 23]. The fine mapping of SNPs showing significant association with Italian PC1 gradient and of SNPs showing |iHS| values exceeding the threshold, together with the genomic intervals reported to be adaptation targets by previous studies, allowed us to define a core-region of 209 kb (chromosome 13, position 74,863,339–75,072,592) in which North–South differentiation and positive selection signatures were clustered (Fig. 3a). The core-region contained a single validated RefSeq gene encoding for a lincRNA (*LINC00381*) with no functional information available. According to UCSC [24] annotation, a second gene (AX747962) transcribed from the opposite strand was present (Fig. 3b). The annotation of lincRNAs based on the work by Cabili et al. (2011) [25] confirmed the existence of this transcript and suggested the presence of a third transcriptional unit (*TCONS_00022202)* giving rise to two alternative spliced isoforms with high expression levels in white blood cells and in lymph nodes. Data from the ENCODE project [26] supported the transcriptional activity of this region in a Normal Human Epidermal Keratinocyte (NHEK) cell line, where an RNAseq peak, an enrichment of histone H3 acetylation on lysine 27 (H3K27Ac) and histone H3 mono-methylation on lysine 4 (H3K4Me1), together with a cluster of DNaseI hypersensitive sites were demonstrated in the region. The 209 kb core-region also contained a SNP (rs17714988; position 74,995,660) reported as associated with cytokine responses in smallpox vaccine recipients [27]. When analyzed at haplotypic level, the rs17714988 allele, correlated with a higher level of secreted IFNα, was found on the haplotype containing the alleles for which we demonstrated both positive selection and association with the Italian latitudinal gradient.

## Discussion

In this study, we investigated the genomic loci contributing to the genetic latitudinal gradient of the Italian population at genome-wide level. By Principal Component Analysis we identified the North–South gradient as the main axis of the Italian genetic variation in agreement with other studies [5, 7, 9]. Our results are slightly different from the previous work of Di Gaetano and co-authors that investigated the genetic structure of the Italian population using genome-wide markers because it identified the PC1 as the one separating Sardinia from the rest of Italy and the PC2 as latitude-related [9]. However, while in our study the proportion of Sardinian samples reflected that observed in the Italian population, in the study by Di Gaetano et al. Sardinian samples were over-represented, probably causing the differences in the results. In our study, we used the analysis of surnames to establish a correlation between PC1 and latitude. We are aware that our approach has some limitations. In particular, the use of surnames to define the geographic origin of samples can misclassify some individuals because it does not take into account the maternal contribution. However, since we used the origin information after the PCA to interpret this result our choice did not alter the subsequent analyses, which were only based on genetic data. Furthermore, similar results would have been obtained using the place of birth or the place of residence but they were only partly available for our samples and we could not make comparisons about their usefulness.

On the basis of the SNPs significantly contributing to the first Italian principal component, we identified 210 genomic loci that we considered as the main contributors to the North–South gradient. The evaluation of the loci by an interval-based enrichment approach revealed us that they were not randomly located in the genome but preferentially spanned genic regions and, in particular, regions containing protein coding genes. Moreover, the identified loci resulted enriched in disease-associated genes and risk-variants underlining the functional relevance of these regions. Within the most associated loci, genomic regions known for their contribution to the European genetic diversity were contained such as the *LCT, HERC2/OCA2* and *HLA* regions [28, 29]. The human pigmentation genetic diversity, showing a latitudinal gradient shaped by natural selection due to light exposure, was largely contributing to the Italian North–South gradient. Indeed, in addition to *HERC2*/*OCA2*, other pigmentation genes as *SLC45A2* [30], *HPS5* [31] and *EXOC2-IRF4* [32] were found in PC1-associated loci. Interestingly, it was recently reported that the positively selected gene SLC45A2 was also associated with melanoma susceptibility in a South European population, thus underlining the important link between selection and diseases [33]. In addition to these specific examples, the important role of pigmentation emerged also from the gene-set enrichment analysis together with the immune response, another biological function known to be target of recent selection [23]. The Gene Ontology analysis for Biological Process showed an enrichment of genes involved in signal transduction and in particular of membrane receptors triggering the immune cascade like the Toll-like receptors (*TLR1*, *TLR6*, *TLR10*) or *CD79A*, a subunit of the B-cell antigen receptor. In agreement with this result, the Gene Ontology analysis for Cellular Component revealed an enrichment of genes acting at the plasma membrane level, thus modulating cell behavior in response to external stimuli. The enrichment of genes with a role in the immune system emerged even more clearly from the analysis of canonical pathways. The pathways identified by the IPA and MSignDB analyses highlighted different aspects of the immune response. The majority of them converged to the Nf-kB signaling as previously suggested [23, 34, 35] and several genes encoding for its components (i.e. *CHUK*, *NFKBIA*) or regulators (i.e. *IGF1R*, *UBE2V*, *HRAS*, *PIK32C2G*, *TLRs*) were located in the Italian PC1-associated loci. Taken together, these data pointed out the immune response as the biological process mainly differentiated along the Italian peninsula, probably as a preferential target of natural selection.

The most likely explanation for the contribution of immunity to population differentiation is its function in host defense against pathogens [36, 37]. In fact, several of the genes that contribute to the Italian population structure were described as involved in infectious disease susceptibility or resistance. For example, malaria, which was endemic in the Mediterranean area and especially in Italy [38, 39] emerged as an infectious disease which had a great impact on the Italian genetic diversity. *HBB,* a gene known to harbor alleles conferring protection against malaria and to be a target of balancing selection [40], is among the genes showing strong differentiation in our dataset. Moreover, the complement factor 1 (*CR1*) gene, suggested to be involved in malaria susceptibility [41], and the *FCGR2B* gene, demonstrated to harbor malaria protective alleles [42, 43], were also identified by our analysis, thus strengthening the mark of malaria in the Italian genome.

Malaria was not the only pathology for which we recognized traces in the Italian population. The Toll-like receptor gene cluster, shown to modulate the response to *Yersinia pestis* [44] and its member *TLR1* involved in leprosy susceptibility [45], were also identified as well as the *IFITM3* gene, whose expression was demonstrated to protect against influenza A infection [46]. Furthermore, a region on chromosome 2 (locus 22 in Additional file 7), recently described as positively selected as a consequence of adaptation to *Vibrio cholera* [35], resulted linked to the PC1 trait and subjected to positive selection in our analysis (Additional file 9).

Recent studies highlighted the presence of adaptation signals in non-coding regions likely owing regulatory functions [37, 47]. In this regard, the locus on chromosome 13, which we demonstrated correlated to the Italian PC1 trait and subjected to positive selection, appeared particularly interesting as it contains only three lincRNAs transcripts. Among them, the *TCONS_00022202* transcript appeared as the best candidate to exert its role in the immune system, because it is mainly expressed in lymph nodes and white blood cells. The transcriptional activity of the *TCONS_00022202* locus was further supported by recent data provided by the ENCODE project demonstrating that it is enriched in an enrichment in modifications typical of active chromatin and is highly transcribed in the NHEK cell line. The NHEK cell line derived from primary epidermal keratinocytes which represent an effective barrier to the entry of infectious agents and play an active role in the initiation of the immune response. These cells produce a variety of cytokines, growth factors, interleukins and antimicrobial peptides thus representing a cell model to investigate inflammation and immune response. Given that non-coding RNAs are emerging as important regulators of gene expression in the immune response [48, 49], we suggested that the *TCONS_00022202* transcript may represent a new immune-related molecule deserving further investigations.

Intriguingly, a polymorphism located about 22 kb upstream to the *TCONS_00022202* lincRNA was associated with the IFNα response in smallpox vaccine recipients, a phenotype that resembles the host response to the virus. Since the allele correlating with higher level of interferon expression was on the positively selected haplotype, we proposed that the observed signature of positive selection is the effect of adaptation to *Variola virus*. Moreover, the observation that 2 other SNPs (rs17070309 and rs12256830) associated with smallpox-induced cytokine response [27] are located within the Italian PC1-associated loci (loci 104 and 128 in Additional file 7), reinforced the hypothesis that smallpox virus could have shaped the Italian genome diversity.

## Conclusions

In conclusion, our study provides new insights into the Italian population structure by characterizing the main determinants of the current genetic diversity and results in the identification of immunity as the main biological process responsible for genetic differentiation in Italy positive selection target, likely triggered by infective agents. Interestingly, recent studies suggested an important role of loci involved in host defense against pathogens also in autoimmune disease susceptibility. For example, it was proposed that the genetic architecture of inflammatory bowel disease was shaped by pathogen-driven selection [50, 51]. Further investigations are required for a better comprehension of evolutionary processes and their relationship with disease predisposition.

## Methods

All the reported genomic coordinates were based on the February 2009 assembly of the human genome (hg19/GRCh37). The statistical analyses, unless otherwise specified, were performed with R, version 2.15.3 [52].

### Study samples and genotyping

Before the quality control procedure a total of 1736 individuals was available for this study. In particular, 1648 individuals of self-reported Italian origin, recruited in North Italy had surname information accessible. Their genotype data were assembled from a study of cerebrovascular disease including 697 cases and 951 controls. Controls were recruited among blood donors and volunteer healthy people, 409 already analyzed in a study on obesity and 392 in the PROCARDIS study [53]. All individuals were enrolled in the study following written informed consent and ethical approval from the institutional review boards for each sample collection, namely Ethics Committee of the Fondazione IRCCS Istituto Neurologico Carlo Besta, Istituto Auxologico Italiano and Lombardy Region. 88 samples from the Tuscan cohort (TSI) genotyped in the HapMap project phase III [54] were added to the study cohort, for a total of 1736 Italian individuals. For the evaluation of the genetic variability of the Italian population in the context of European and Mediterranean populations, we analyzed genotypic data of 303 individuals drawn from the Human Genome Diversity Project (HGDP [55]), 186 individuals from the Behar et al., 2010 study [56], 50 individuals from McEvoy et al., 2009 [57] and 25 individuals from the Wellcome Trust Case Control Consortium—WTCCC [58] (Additional file 2).

### Surname-based definition of individual’s geographical origin

The geographic origin of individuals was defined through the analysis of their surnames. The birth place and the place of residence were not available for all the individuals and previous analyses demonstrated that they are not suitable to infer the individual’s geographic origin because of recent migrations [59]. In Italy surnames are transmitted patrilineally and can be considered as Y-chromosome genetic markers. For this reason we used the surname analysis as a tool to infer the place of origin. In particular, our surname analysis was based on the Italian Surnames database that was established extracting data from the complete national telephone directory of year 1993 (18,554,688 subscribers corresponding of about 33 % of the whole Italian population) and includes a total of 332,525 different surnames together with their frequencies in the different Italian administrative zones [60]. A supervised frequency-based approach combined with linguistic and historical records was used to analyze surnames and to determine their putative geographical origin. For the purposes of this study, the Italian territory was subdivided into four main areas: North (comprising 8 administrative regions, namely: Piedmont, Aosta Valley, Lombardy, Liguria, Veneto, Trentino Alto Adige, Friuli Venezia Giulia and Emilia Romagna), Central (comprising 5 administrative regions, namely: Tuscany, Marche, Umbria, Lazio and Abruzzo), South (comprising 6 administrative regions, namely: Molise, Campania, Apulia, Basilicata, Calabria and Sicily) and Sardinia. The analysis of surnames frequency distribution combined in the four main areas allowed to assign a geographical origin to a total of 1238 individuals. The remaining 410 individuals (25 %) had a surnames whose geographical origin could not be unambiguously assigned [60]. The surnames analysis was conducted independently and anonymously from the genotypic analyses and the match of data was conducted by authorized personnel. The 88 individuals from the TSI cohort of HapMap were assigned to Central Italy based on their reported origin.

### Genotype data analysis and quality control procedures

Managing of genotype data and quality control procedures were performed with PLINK 1.0.7 [61]. For the Italian dataset a total of 487,999 SNPs was initially available for the analyses. The quality control procedure resulted in the exclusion of 35,003 markers with minor allele frequency below 0.05, 4100 markers for genotyping rate below 0.97 and 21 individuals for genotype call below 0.97. Because LD features could distort the PCA analysis, one member of each pair of SNPs with *r*^2^ greater than 0.4, in windows of 200 SNPs (sliding window of 25 SNPs), was removed using the indep-pairwise command in PLINK. After the quality control procedure, a total of 1715 individuals and 172,111 SNPs was considered for the analysis. Finally, 1000 SNPs from the 8p23.1 genetic region, known to harbor a large inversion polymorphism [62, 63], were excluded because they could distort the subsequent analyses. On the dataset used to calculate the iHS statistics we did not exclude the SNPs highly correlated. The quality control procedure applied to the Mediterranean dataset is described in the Additional file 3.

### Principal component analysis and ancestry estimation

Principal Component Analysis (PCA) was carried out using the *smartpca* tool of the EIGENSOFT package version 3.0 using the default parameter and no outlier exclusion [16]. The correlation between PC1 and latitude was tested using the R cor.test function. To each individual we attributed the latitude value corresponding to the capital of the administrative region identified as individual place of origin.

The SNPs significantly associated with the first principal component were identified through a linear regression model in PLINK. PC1 was used as a response variable and the SNP as the explanatory one. The analysis of SNPs associated with Italian PC2 identified a small number of significant SNPs, likely because samples from Sardinia were too few. For this reason PC2 was not further examined. To infer the ancestry proportions in the European/Mediterranean dataset we applied the unsupervised clustering algorithm ADMIXTURE [17]. The analysis was repeated from *K* = 2 to *K* = 7. The optimal number of K was estimated through the cross-validation procedure using the --cv = 10 option.

### Test for selection

The presence of signal of selection was tested using the iHS statistics [21], calculated using the R package *rehh* [64]. This test detected the presence of extended haplotypes surrounding each core SNP to identify candidate alleles for selective sweeps. Before running the analysis, the genotypes, not LD-pruned, were phased using *fastPHASE* [65]. For each SNP the ancestral state was identified from NCBI dbSNP (build 139) and the genetic position along the chromosomes was taken from the HapMap Consortium (release 22, B36). To determine the significant regions the SNP’s iHS scores were grouped in non overlapping 200 kb windows and for each window we calculated the fraction of SNPs with an |iHS| > = 2. The windows with a total number of SNPs less than 20 were excluded from the analysis. The fraction of windows in the top 5 % tail of iHS distribution was considered as significant.

The comparison with literature data was performed selecting articles reporting genome-wide analyses of positive natural selection carried out on reference populations belonging to the HapMap project or to the Human Genome Diversity Project, published up to 2013.

### Loci definition

The genomic intervals corresponding to each PC1-associated SNP were defined on the basis of the LD feature of the genome through the Gene Relationships Across Implicated Loci (GRAIL) tool [66]. The tool was run using the list of 270 significant SNPs and the HapMap CEU (release 22) as a reference population. The genes overlapping the regions were defined from the UCSC RefSeq Genes track [67].

### Enrichment analyses

The interval-based enrichment tests were performed with INRICH v.1.0 [68] using 1,000,000 permutations both in the first and in the second phase of the analysis. Specifically, protein coding genes, ncRNAs and pseudogenes were defined according to the NCBI Gene database (http://www.ncbi.nlm.nih.gov/gene/) limiting the query to RefSeq records. The list of genes involved in Mendelian diseases was defined filtering the Online Mendelian Inheritance in Man (http://omim.org/) catalogue to exclude unconfirmed diseases, traits not involved in disorders and inconsistent or tentative records. The list of SNPs associated with complex disorders was retrieved from the NHGRI GWAS catalogue (http://www.genome.gov/gwastudies/; date accessed on April, 3rd 2014) [19]. The manually curated list of genes involved in pathways known to be target of positive selection was downloaded from the Composite of Multiple Signals website (http://www.broadinstitute.org/mpg/cms) [23].

The gene-set enrichment analysis was performed using the MSigDB tool of the GSEA package (http://www.broadinstitute.org/gsea/msigdb/index.jsp) querying Gene Ontology as source annotation database and considering the categories with a corrected *p*-value less than 1 × 10^−4^. The canonical pathways were investigated using QIAGEN’s Ingenuity® Pathway Analysis (IPA®, QIAGEN Redwood City, www.qiagen.com/ingenuity) and the first 10 significant canonical pathways were reported.

The functional categories reported in Fig. 2 were generated as follows. The immune category was defined combining evidence of genes involved in the immune system from the Immune System term of Gene Ontology Biological Process, the immune-related IPA and Reactome canonical pathways. The autoimmunity category was defined from the presence of association signals with autoimmune diseases. The host-pathogen category was manually defined exploiting the information from NCBI Gene database and literature confirmation.

### Availability of data and materials

The list of the 210 loci associated with Italian population PC1 is available in Additional file 7. For each of the 210 loci, the genomic coordinates and the identifiers of the associated SNPs are provided.

## Additional files

Additional file 1:
**Geographical distribution of Italian samples based on surnames.** (DOCX 70 kb) Additional file 2:
**The European/Mediterranean dataset.** (DOCX 97 kb) Additional file 3:
**Analysis of the Italian dataset with European and Mediterranean populations.** (DOCX 149 kb) Additional file 4:
**PCA of European and Mediterranean populations.** (A) Plot of the first two principal components of the Italian population combined with populations from continental Europe and Mediterranean area; (B) geographical localization of the analyzed samples. Legend of symbols and colors used is reported below. The map of European/Mediterranean area was obtained plotting a suitable portion of the spatial world data downloaded from http://thematicmapping.org/. (PDF 1212 kb) Additional file 5:
**Graphical representation of ADMIXTURE analysis results.** The analysis was performed assuming 4 ancestral populations (*K* = 4) and including all the samples used for Principal Component Analysis in the European/Mediterranean dataset. The populations with an asterisk (*) are those of the present study. (PDF 147 kb) Additional file 6:
**PCA of the Italian dataset using the 270** **PC1-associated** **SNPs.** Plot of the first two principal components showing that the 270 SNPs recreate the genetic latitudinal gradient observed in Italy. (PDF 73 kb) Additional file 7:
**Features of the 210 Italian** **PC1-associated** **loci.** (XLSX 47 kb) Additional file 8:
**Gene-enrichment analyses. **The genes present in the enriched GO Biological Process categories, Cellular Component categories and IPA canonical pathways are reported. (XLSX 57 kb) Additional file 9:
**UCSC genome browser view of the locus 22 of Additional file** 7 **.** (A) In this panel is showed the genomic region chr2:95965626–97094126. In particular are reported: the PC1 association signals (vertical dark violet lines), the |iHS| value for each tested SNP (vertical dark green), the genomic region previously identified as positively selected by Karlsson et al. (2013) [35] (horizontal green bar) and the two genomic intervals resulted positively selected in our analysis (horizontal red bars); (B) detail of the identified positively selected region showing the RefSeq genes contained. (PDF 113 kb)

## Abbreviations

GO: Gene Ontology
GRAIL: Gene Relationships Across Implicated Loci
GSEA: Gene Set Enrichment Analysis
GWAS: Genome Wide Association Study
H3K4Me1: Histone H3 mono-methylation on lysine 4
H3K27Ac: Histone H3 acetylation on lysine 27
HGDP: Human Genome Diversity Projects
HGNC: HUGO Gene Nomenclature Committee
HLA: Human leukocyte antigen
iHS: Integrated haplotype score
IPA: Ingenuity Pathway Analysis
LD: Linkage disequilibrium
lincRNA: Long intergenic non-coding RNA
NHEK: Normal Human Epidermal Keratinocyte
NHGRI: National Human Genome Research Institute
PCA: Principal Component Analysis
PCs: Principal Components
SNPs: Single nucleotide polymorphisms
WTCCC: Wellcome Trust Case Control Consortium

## References

[1] Myles S, Tang K, Somel M, Green RE, Kelso J, Stoneking M (2008). Identification and analysis of genomic regions with large between-population differentiation in humans. Ann Hum Genet.

[2] Moonesinghe R, Ioannidis JP, Flanders WD, Yang Q, Truman BI, Khoury MJ (2012). Estimating the contribution of genetic variants to difference in incidence of disease between population groups. Eur J Hum Genet.

[3] Corona E, Chen R, Sikora M, Morgan AA, Patel CJ, Ramesh A (2013). Analysis of the genetic basis of disease in the context of worldwide human relationships and migration. PLoS Genet.

[4] Raj T, Kuchroo M, Replogle JM, Raychaudhuri S, Stranger BE, De Jager PL (2013). Common risk alleles for inflammatory diseases are targets of recent positive selection. Am J Hum Genet.

[5] Novembre J, Johnson T, Bryc K, Kutalik Z, Boyko AR, Auton A (2008). Genes mirror geography within Europe. Nature.

[6] Lao O, Lu TT, Nothnagel M, Junge O, Freitag-Wolf S, Caliebe A (2008). Correlation between genetic and geographic structure in Europe. Curr Biol.

[7] Nelis M, Esko T, Mägi R, Zimprich F, Zimprich A, Toncheva D (2009). Genetic structure of Europeans: A view from the North-East. PLoS One.

[8] Cavalli-Sforza LL, Menozzi P, Piazza A (1994). The history and geography of human genes.

[9] Di Gaetano C, Voglino F, Guarrera S, Fiorito G, Rosa F, Di Blasio AM (2012). An overview of the genetic structure within the Italian population from genome-wide data. PLoS One.

[10] Soares P, Achilli A, Semino O, Davies W, Macaulay V, Bandelt HJ (2010). The archaeogenetics of Europe. Curr Biol.

[11] Akey JM (2009). Constructing genomic maps of positive selection in humans: Where do we go from here?. Genome Res.

[12] Scheinfeldt LB, Tishkoff SA (2013). Recent human adaptation: Genomic approaches, interpretation and insights. Nat Rev Genet.

[13] Bersaglieri T, Sabeti PC, Patterson N, Vanderploeg T, Schaffner SF, Drake JA (2004). Genetic signatures of strong recent positive selection at the lactase gene. Am J Hum Genet.

[14] Pritchard JK, Pickrell JK, Coop G (2010). The genetics of human adaptation: Hard sweeps, soft sweeps, and polygenic adaptation. Curr Biol.

[15] Turchin MC, Chiang CW, Palmer CD, Sankararaman S, Reich D, Hirschhorn JN (2012). Evidence of widespread selection on standing variation in Europe at height-associated SNPs. Nat Genet.

[16] Patterson N, Price AL, Reich D (2006). Population structure and eigenanalysis. PLoS Genet.

[17] Alexander DH, Novembre J, Lange K (2009). Fast model-based estimation of ancestry in unrelated individuals. Genome Res.

[18] Gray K, Daugherty L, Gordon S, Seal R, Wright M, Bruford E (2013). Genenames.org: The HGNC resources in. Nucleic Acids Res.

[19] Welter D, MacArthur J, Morales J, Burdett T, Hall P, Junkins H (2014). The NHGRI GWAS Catalog, a curated resource of SNP-trait associations. Nucleic Acids Res.

[20] Subramanian A, Tamayo P, Mootha V, Mukherjee S, Ebert B, Gillette M (2005). Gene set enrichment analysis: A knowledge-based approach for interpreting genome-wide expression profiles. Proc Natl Acad Sci U S A.

[21] Voight BF, Kudaravalli S, Wen X, Pritchard JK (2006). A map of recent positive selection in the human genome. PLoS Biol.

[22] Pickrell JK, Coop G, Novembre J, Kudaravalli S, Li JZ, Absher D (2009). Signals of recent positive selection in a worldwide sample of human populations. Genome Res.

[23] Grossman SR, Andersen KG, Shlyakhter I, Tabrizi S, Winnicki S, Yen A (2013). Identifying recent adaptations in large-scale genomic data. Cell.

[24] Kent W, Sugnet C, Furey T, Roskin K, Pringle T, Zahler A (2002). The human genome browser at UCSC. Genome Res.

[25] Cabili M, Trapnell C, Goff L, Koziol M, Tazon-Vega B, Regev A (2011). Integrative annotation of human large intergenic noncoding RNAs reveals global properties and specific subclasses. Genes Dev.

[26] Dunham I, Kundaje A, Aldred S, Collins P, Davis C, Doyle F (2012). An integrated encyclopedia of DNA elements in the human genome. Nature.

[27] Kennedy RB, Ovsyannikova IG, Pankratz VS, Haralambieva IH, Vierkant RA, Poland GA (2012). Genome-wide analysis of polymorphisms associated with cytokine responses in smallpox vaccine recipients. Hum Genet.

[28] Heath S, Gut I, Brennan P, McKay J, Bencko V, Fabianova E (2008). Investigation of the fine structure of European populations with applications to disease association studies. Eur J Hum Genet.

[29] Donnelly MP, Paschou P, Grigorenko E, Gurwitz D, Barta C, Lu RB (2012). A global view of the OCA2-HERC2 region and pigmentation. Hum Genet.

[30] Lucotte G, Mercier G, Dieterlen F, Yuasa I (2010). A Decreasing Gradient of 374 F Allele Frequencies in the Skin Pigmentation Gene SLC45A2, from the North of West Europe to North Africa. Biochem Genet.

[31] Zhang Q, Zhao B, Li W, Oiso N, Novak E, Rusiniak M (2003). Ru2 and Ru encode mouse orthologs of the genes mutated in human Hermansky-Pudlak syndrome types 5 and 6. Nat Gen.

[32] Praetorius C, Grill C, Stacey SN, Metcalf AM, Gorkin DU, Robinson KC (2013). A polymorphism in IRF4 affects human pigmentation through a tyrosinase-dependent MITF/TFAP2A pathway. Cell.

[33] López S, García O, Yurrebaso I, Flores C, Acosta-Herrera M, Chen H (2014). The interplay between natural selection and susceptibility to melanoma on allele 374 F of SLC45A2 gene in a South European population. PLoS One.

[34] Kamberov Y, Wang S, Tan J, Gerbault P, Wark A, Tan L (2013). Modeling recent human evolution in mice by expression of a selected EDAR variant. Cell.

[35] Karlsson E, Harris J, Tabrizi S, Rahman A, Shlyakhter I, Patterson N (2013). Natural selection in a Bangladeshi population from the Cholera-Endemic Ganges River Delta. Sci Transl Med.

[36] Fumagalli M, Sironi M, Pozzoli U, Ferrer-Admettla A, Pattini L, Nielsen R (2011). Signatures of environmental genetic adaptation pinpoint pathogens as the main selective pressure through human evolution. Plos Genet.

[37] Karlsson E, Kwiatkowski D, Sabeti P (2014). Natural selection and infectious disease in human populations. Nat Rev Gen.

[38] Cavalli-Sforza LL, Bodmer WF (1971). The Genetics of Human populations.

[39] Majori G (2012). Short history of malaria and its eradication in Italy with short notes on the fight against the infection in the mediterranean basin. Mediterr J Hematol Infect Dis.

[40] Mangano VD, Modiano D (2014). An evolutionary perspective of how infection drives human genome diversity: The case of malaria. Curr Opin Immunol.

[41] Stoute J (2011). Complement receptor 1 and malaria. Cell Microbiol.

[42] Clatworthy M, Willcocks L, Urban B, Langhorne J, Williams T, Peshu N (2007). Systemic lupus erythematosus-associated defects in the inhibitory receptor Fc gamma RIIb reduce susceptibility to malaria. Proc Natl Acad Sci U S A.

[43] Willcocks L, Carr E, Niederer H, Rayner T, Williams T, Yang W (2010). A defunctioning polymorphism in FCGR2B is associated with protection against malaria but susceptibility to systemic lupus erythematosus. Proc Natl Acad Sci U S A.

[44] Laayouni H, Oosting M, Luisi P, Ioana M, Alonso S, Ricano-Ponce I (2014). Convergent evolution in European and Rroma populations reveals pressure exerted by plague on Toll-like receptors. Proc Natl Acad Sci U S A.

[45] Wong S, Gochhait S, Malhotra D, Pettersson F, Teo Y, Khor C (2010). Leprosy and the adaptation of human toll-like receptor 1. Plos Pathog.

[46] Everitt A, Clare S, Pertel T, John S, Wash R, Smith S (2012). IFITM3 restricts the morbidity and mortality associated with influenza. Nature.

[47] Fumagalli M, Sironi M (2014). Human genome variability, natural selection and infectious diseases. Curr Opin Immunol.

[48] Atianand MK, Fitzgerald KA (2014). Long non-coding RNAs and control of gene expression in the immune system. Trends Mol Med.

[49] Heward JA, Lindsay MA (2014). Long non-coding RNAs in the regulation of the immune response. Trends Immunol.

[50] Jostins L, Ripke S, Weersma R, Duerr R, McGovern D, Hui K (2012). Host-microbe interactions have shaped the genetic architecture of inflammatory bowel disease. Nature.

[51] Quintana-Murci L, Clark A (2013). Population genetic tools for dissecting innate immunity in humans. Nat Rev Immunol.

[52] Team RC (2013). R: A language and environment for statistical computing.

[53] Peden J, Hopewell J, Saleheen D, Chambers J, Hager J, Soranzo N (2011). A genome-wide association study in Europeans and South Asians identifies five new loci for coronary artery disease. Nat Gen.

[54] Altshuler D, Gibbs R, Peltonen L, Dermitzakis E, Schaffner S, Yu F (2010). Integrating common and rare genetic variation in diverse human populations. Nature.

[55] Li J, Absher D, Tang H, Southwick A, Casto A, Ramachandran S (2008). Worldwide human relationships inferred from genome-wide patterns of variation. Science.

[56] Behar D, Yunusbayev B, Metspalu M, Metspalu E, Rosset S, Parik J (2010). The genome-wide structure of the Jewish people. Nature.

[57] McEvoy B, Montgomery G, McRae A, Ripatti S, Perola M, Spector T (2009). Geographical structure and differential natural selection among North European populations. Genome Res.

[58] Burton P, Clayton D, Cardon L, Craddock N, Deloukas P, Duncanson A (2007). Genome-wide association study of 14,000 cases of seven common diseases and 3,000 shared controls. Nature.

[59] Zei G, Lisa A, Fiorani O, Magri C, Quintana-Murci L, Semino O (2003). From surnames to the history of Y chromosomes: The Sardinian population as a paradigm. Eur J Hum Genet.

[60] Boattini A, Lisa A, Fiorani O, Zei G, Pettener D, Manni F (2012). General Method to Unravel Ancient Population Structures through Surnames, Final Validation on Italian Data. Hum Biol.

[61] Purcell S, Neale B, Todd-Brown K, Thomas L, Ferreira MA, Bender D (2007). PLINK: A tool set for whole-genome association and population-based linkage analyses. Am J Hum Genet.

[62] Deng L, Zhang Y, Kang J, Liu T, Zhao H, Gao Y (2008). An unusual haplotype structure on human chromosome 8p23 derived from the inversion polymorphism. Hum Mutat.

[63] Tian C, Plenge RM, Ransom M, Lee A, Villoslada P, Selmi C (2008). Analysis and application of European genetic substructure using 300 K SNP information. PLoS Genet.

[64] Gautier M, Vitalis R (2012). rehh: An R package to detect footprints of selection in genome-wide SNP data from haplotype structure. Bioinformatics.

[65] Scheet P, Stephens M (2006). A fast and flexible statistical model for large-scale population genotype data: Applications to inferring missing genotypes and haplotypic phase. Am J Hum Genet.

[66] Raychaudhuri S, Plenge R, Rossin E, Ng A, Purcell S, Sklar P (2009). Identifying relationships among genomic disease regions: Predicting genes at pathogenic snp associations and rare deletions. Plos Genet.

[67] Pruitt K, Tatusova T, Maglott D (2007). NCBI reference sequences (RefSeq): A curated non-redundant sequence database of genomes, transcripts and proteins. Nucleic Acids Res.

[68] Lee PH, O’Dushlaine C, Thomas B, Purcell SM (2012). INRICH: Interval-based enrichment analysis for genome-wide association studies. Bioinformatics.

