# Supplementary material for: Characterization of the biological processes shaping the genetic structure of the Italian population
Source: BMC Genet. 2015 Nov 9;16:132. doi: 10.1186/s12863-015-0293-x (PMC4640365; doi:10.1186/s12863-015-0293-x)
Supplement: Additional file 1: — Geographical distribution of Italian samples based on surnames. (DOCX 70 kb) [file 12863_2015_293_MOESM1_ESM.docx]

**Additional file 1: Geographical distribution of Italian samples based on surnames analysis**

| **macro area** | **region** | **N** |
| --- | --- | --- |
| North | Emilia-Romagna | 52 |
| North | Friuli-Venezia Giulia | 13 |
| North | Liguria | 12 |
| North | Lombardy | 264 |
| North | Piedmont | 120 |
| North | Trentino-Alto Adige | 3 |
| North | Veneto | 89 |
| North | Not Defined^(1)^ | 97 |
| ***Total North*** | | **650** |
| Center | Abruzzo | 13 |
| Center | Lazio | 12 |
| Center | Marche | 10 |
| Center | Tuscany | 33 |
| Center | Umbria | 7 |
| Center | Not Defined^(1)^ | 25 |
| ***Total Center*** | | **100** |
| South | Basilicata | 5 |
| South | Calabria | 53 |
| South | Campania | 65 |
| South | Molise | 4 |
| South | Apulia | 85 |
| South | Sicily | 127 |
| South | Not Defined^(1)^ | 124 |
| ***Total South*** | | **463** |
| Sardinia | Sardinia | 25 |
| ITALY | Not Defined^(2)^ | 389 |

^(1)^ individuals whose origin resulted not defined at regional level but defined at macro area level.

^(2)^ individuals whose origin resulted not defined at any level.
