# Supplementary material for: Characterization of the biological processes shaping the genetic structure of the Italian population
Source: BMC Genet. 2015 Nov 9;16:132. doi: 10.1186/s12863-015-0293-x (PMC4640365; doi:10.1186/s12863-015-0293-x)
Supplement: Additional file 2: — The European/Mediterranean dataset. (DOCX 97 kb) [file 12863_2015_293_MOESM2_ESM.docx]

| **Geographic Region** | **Population** | **Source** | **Number of individuals** |
| --- | --- | --- | --- |
| Italy | NORTHERN ITALIANS | present Study | 647 |
| Italy | Northern Italians | Li et al., 2009 | 13 |
| Italy | CENTRAL ITALIANS | present Study | 100 |
| Italy | TSI | HapMap 3 | 84 |
| Italy | Tuscans | Li et al., 2009 | 8 |
| Italy | SOUTHERN ITALIANS | present Study | 460 |
| Italy | SARDINIANS | present Study | 25 |
| Italy | Sardinians | Li et al., 2009 | 28 |
| Europe | Cypriots | Behar et al., 2010 | 12 |
| Europe | English | WTCCC | 25 |
| Europe | Finnish | McEvoy et al., 2009 | 25 |
| Europe | French | Li et al., 2009 | 28 |
| Europe | Hungarians | Behar et al., 2010 | 19 |
| Europe | Lithuanians | Behar et al., 2010 | 10 |
| Europe | Romanians | Behar et al., 2010 | 16 |
| Europe | Russians | Li et al., 2009 | 25 |
| Europe | Spaniards | Behar et al., 2010 | 12 |
| Europe | Swedish | McEvoy et al., 2009 | 25 |
| Caucasus | Armenians | Behar et al., 2010 | 19 |
| Caucasus | Georgians | Behar et al., 2010 | 20 |
| Middle East | Bedouins | Li et al., 2009 | 18 |
| Middle East | Druze | Li et al., 2009 | 19 |
| Middle East | Jordanians | Behar et al., 2010 | 19 |
| Middle East | Palestinians | Li et al., 2009 | 24 |
| Middle East | Syrians | Behar et al., 2010 | 15 |
| Middle East | Turks | Behar et al., 2010 | 19 |
| North Africa | Egyptians | Behar et al., 2010 | 12 |
| North Africa | Moroccans | Behar et al., 2010 | 9 |
| North Africa | Mozabite | Li et al., 2009 | 22 |

**Additional file 2: The European/Mediterranean dataset**
