# Supplementary material for: Characterization of the biological processes shaping the genetic structure of the Italian population
Source: BMC Genet. 2015 Nov 9;16:132. doi: 10.1186/s12863-015-0293-x (PMC4640365; doi:10.1186/s12863-015-0293-x)
Supplement: Additional file 3: — Analysis of the Italian dataset with European and Mediterranean populations. (DOCX 149 kb) [file 12863_2015_293_MOESM3_ESM.docx]

**Analysis of the Italian diversity in the European and Mediterranean context**

To evaluate the Italian genetic diversity in its geographical context we exploited public available genotype data selected from European and Mediterranean populations surrounding Italy. In this regard, a second dataset was established combining the previous Italian samples with: 197 European individuals from different countries, 39 individuals from Caucasus, 114 individuals from the Middle East area and 43 individuals from North Africa (see Additional File 2). Among the Europeans, 25 English, Finnish and Swedish samples were randomly sampled from all the individuals available in order to keep the number of total samples per population comparable. A total of 49 individuals of Italian origin, available from the Human Genetic Diversity Project (HGDP), were also included as internal controls. The samples were merged using PLINK and the QC thresholds applied were the same of the Italian dataset. We did not observe a clustering of individuals according to the genotyping center, allowing us to exclude major effect of the sample source on the observed variability. As an additional confirmation, we observed that samples from geographical close areas, coming from different studies, clustered together in the PCA plot.

The newly assembled dataset, after quality control and LD pruning procedures, comprised 1758 samples and 134,579 SNPs.

The genetic variability within the European/Mediterranean area was summarized by PCA. The plot of the first two principal components is reported in Additional File 4. The first PC (0.42% of total variance explained) appeared almost centered on the central region of Italy and it mainly separated people of the Mediterranean area from whose of continental Europe. North Africans, Middle Easterners and Caucasian appeared spread along negative PC1 values with Mozabite (n=22; average PC1= -0.08, sd= 0.0076) and Moroccans (n=9; average PC1= -0.08, sd=0.0090) showing the most extreme values. Along with positive PC1 values, Finnish (n=25; average PC1= +0.05, sd=0.0018), Russian (n=25; average PC1= +0.04, sd=0.0021) and Lithuanians (n=10; average PC1= *+*0.05, sd=0.0035) resulted the most distant populations. The second PC (0.21% of total variance explained) revealed a positive relationship with the north-south gradient in its European portion whereas in the other side it separated African from Middle Eastern populations. Northern Italians appeared to be more similar to European continental populations rather than to the Mediterranean ones. On the contrary Southern Italians resulted more similar to Middle Eastern people. These findings were confirmed using the maximum-likelihood based ancestry estimation software ADMIXTURE (Additional File 5).
