# Supplementary material for: Characterization of the biological processes shaping the genetic structure of the Italian population
Source: BMC Genet. 2015 Nov 9;16:132. doi: 10.1186/s12863-015-0293-x (PMC4640365; doi:10.1186/s12863-015-0293-x)
Supplement: Additional file 4: — PCA of European and Mediterranean populations. (A) Plot of the first two principal components of the Italian population combined with populations from continental Europe and Mediterranean area; (B) geographical localization of the analyzed samples. Legend of symbols and colors used is reported below. The map of European/Mediterranean area was obtained plotting a suitable portion of the spatial world data downloaded from http://thematicmapping.org/. (PDF 1212 kb) [file 12863_2015_293_MOESM4_ESM.pdf]

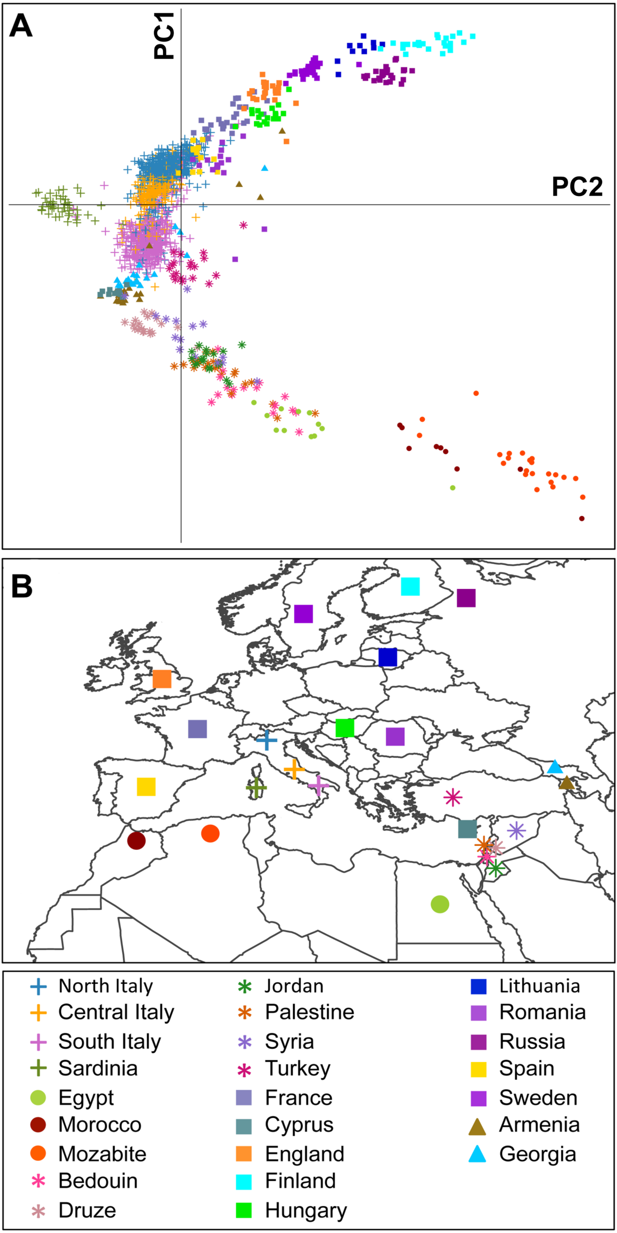
A
PC1
PC2
B
North Italy
Central Italy
South Italy
Sardinia
Egypt
Morocco
Mozabite
Bedouin
Druze
Jordan
Palestine
Syria
Turkey
France
Cyprus
England
Finland
Hungary
Lithuania
Romania
Russia
Spain
Sweden
Armenia
Georgia
