# Supplementary figures and images for: Characterization of the biological processes shaping the genetic structure of the Italian population
Source: BMC Genet. 2015 Nov 9;16:132. doi: 10.1186/s12863-015-0293-x (PMC4640365; doi:10.1186/s12863-015-0293-x)

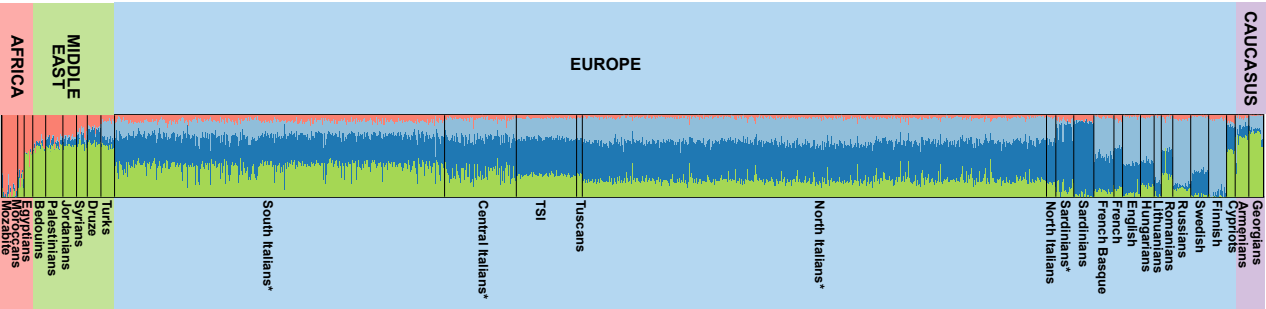

Supplement: Additional file 5: — Graphical representation of ADMIXTURE analysis results. The analysis was performed assuming 4 ancestral populations (K = 4) and including all the samples used for Principal Component Analysis in the European/Mediterranean dataset. The populations with an asterisk (*) are those of the present study. (PDF 147 kb) [file 12863_2015_293_MOESM5_ESM.pdf]
