# Supplementary material for: Characterization of the biological processes shaping the genetic structure of the Italian population
Source: BMC Genet. 2015 Nov 9;16:132. doi: 10.1186/s12863-015-0293-x (PMC4640365; doi:10.1186/s12863-015-0293-x)
Supplement: Additional file 6: — PCA of the Italian dataset using the 270 PC1-associated SNPs. Plot of the first two principal components showing that the 270 SNPs recreate the genetic latitudinal gradient observed in Italy. (PDF 73 kb) [file 12863_2015_293_MOESM6_ESM.pdf]

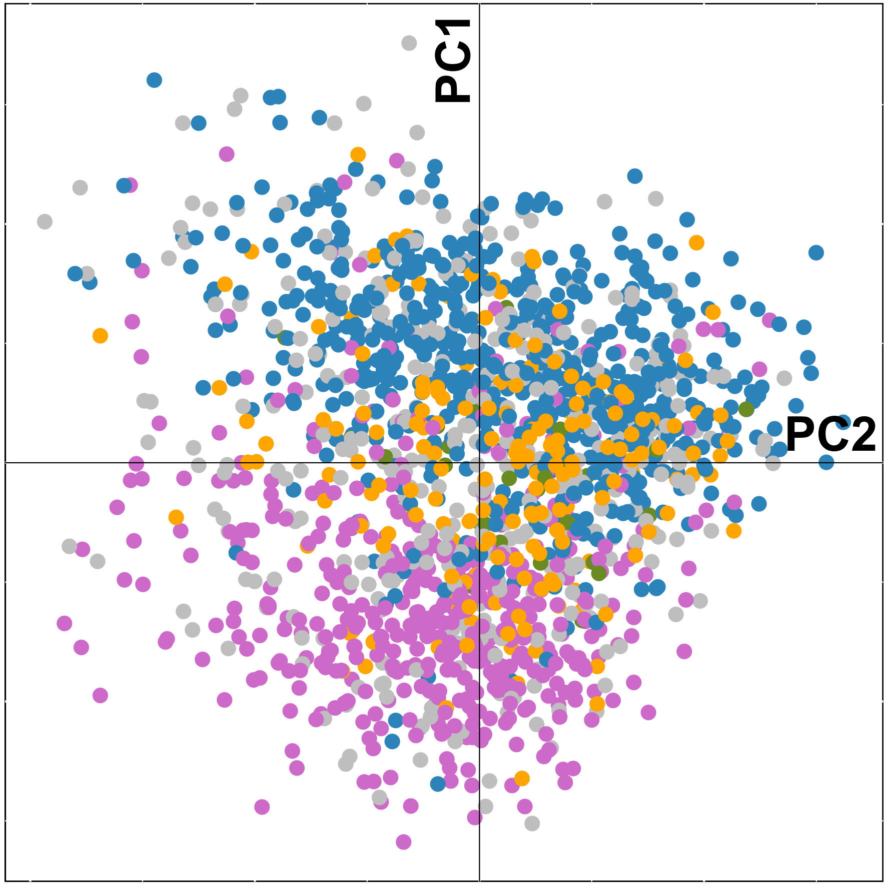

Additional File 6. PCA of the Italian dataset using the 270 PC1-associated SNPs. Plot of the first two principal components showing that the 270 SNPs recreate the genetic latitudinal gradient observed in Italy.
