# Supplementary material for: Characterization of the biological processes shaping the genetic structure of the Italian population
Source: BMC Genet. 2015 Nov 9;16:132. doi: 10.1186/s12863-015-0293-x (PMC4640365; doi:10.1186/s12863-015-0293-x)
Supplement: Additional file 9: — UCSC genome browser view of the locus 22 of Additional file 7 . (A) In this panel is showed the genomic region chr2:95965626–97094126. In particular are reported: the PC1 association signals (vertical dark violet lines), the |iHS| value for each tested SNP (vertical dark green), the genomic region previously identified as positively selected by Karlsson et al. (2013) [35] (horizontal green bar) and the two genomic intervals resulted positively selected in our analysis (horizontal red bars); (B) detail of the identified positively selected region showing the RefSeq genes contained. (PDF 113 kb) [file 12863_2015_293_MOESM9_ESM.pdf]

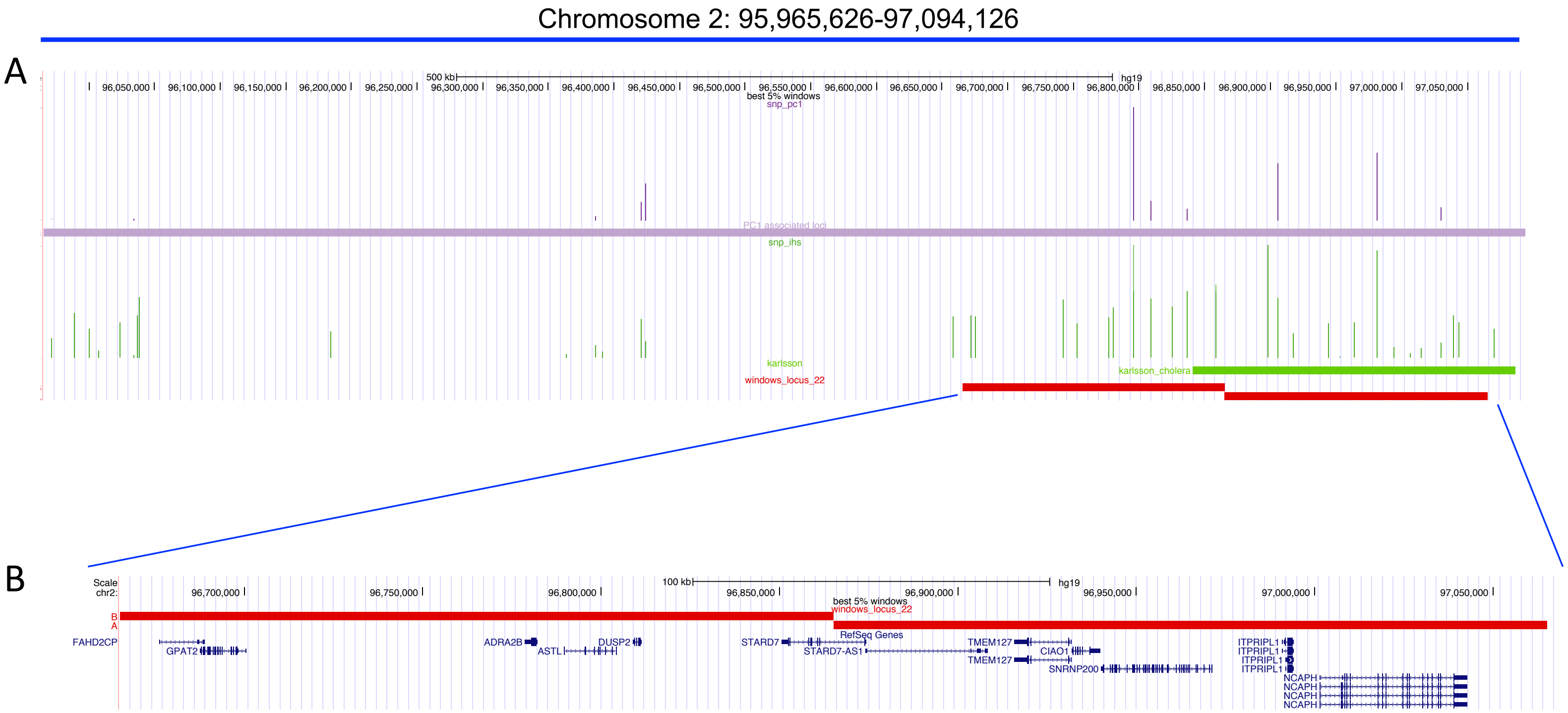

Additional File 9. UCSC genome browser view of the locus 22 in Additional File 7. (A) In this panel is showed the genomic region chr2:95965626-97094126. In particular are reported: the PC1 association signals (vertical dark violet lines), the |iHS| value for each tested SNP (vertical dark green), the genomic region previously identified as positively selected by Karlsson et al. (2013) [29] (horizontal green bar) and the two genomic intervals resulted positively selected in our analysis (horizontal red bars); (B) detail of the identified positively selected region showing the RefSeq genes contained.
